# Practice Variability Combined with Task-Oriented Electromyographic Biofeedback Enhances Strength and Balance in People with Chronic Stroke

**DOI:** 10.1155/2018/7080218

**Published:** 2018-11-26

**Authors:** Peih-Ling Tsaih, Ming-Jang Chiu, Jer-Junn Luh, Yea-Ru Yang, Jiu-Jenq Lin, Ming-Hsia Hu

**Affiliations:** ^1^School and Graduate Institute of Physical Therapy, College of Medicine, National Taiwan University, Taipei, Taiwan; ^2^Department of Physical Therapy, Shu-Tien Urology and Ophthalmology Clinic, Taipei, Taiwan; ^3^Department of Neurology, National Taiwan University Hospital, College of Medicine, Taipei, Taiwan; ^4^National Taiwan University, Taipei, Taiwan; ^5^Department of Physical Therapy and Assistive Technology, National Yang-Ming University, Taipei, Taiwan; ^6^Division of Physical Therapy, Department of Physical Medicine and Rehabilitation, National Taiwan University Hospital, Taipei, Taiwan

## Abstract

**Objectives:**

To investigate the effects of practice variability combined with task-oriented electromyographic biofeedback (EMGBFB) on strength and balance in people with chronic stroke.

**Methods:**

Thirty-three participants were randomly assigned into the constant force EMGBFB tibialis anterior (TA) exercise (constant) group, the variable force EMGBFB tibialis anterior exercise (variable) group, or the upper extremity exercise without EMGBFB (control) group. Subjects in each group received 6 weekly sessions of exercise training (18 sessions, 40 minutes each). Motor outcomes were TA strength, balance (anteroposterior sway amplitude defined by limits of stability test in dynamic posturography), walking speed, Timed Up and Go test (TUGT), and six-minute walk test (6MWT). Data were measured at baseline, 1 day, 2 weeks, and 6 weeks posttraining.

**Results:**

TA strength increased significantly in both the constant and variable groups after training. Balance significantly improved only in the variable group. All participants showed improvements in walking speed, TUGT, and 6MWT.

**Conclusions:**

Task-oriented EMGBFB-assisted TA exercise training improved muscle strength in people with chronic stroke. Practicing to reach varying force levels during EMGBFB-assisted tibialis anterior exercises facilitated improvements in the ability to sway in the anteroposterior direction while standing. Our findings highlight the importance of task-oriented and motor learning principles while using the EMGBFB as an adjunct therapy in stroke rehabilitation. This trial was registered with trial registration number NCT01962662.

## 1. Introduction

Up to 72% of stroke survivors suffer from lower limb weakness [1], and the ankle muscles tend to be more affected than the hip and knee muscles [2]. Force control insufficiency in the tibialis anterior muscle (TA), such as weakness, delayed or decreased recruitment, and reduced motor cortical control, is characterized by an inability to adequately dorsiflex the ankle during functional tasks such as moving from sitting to standing, stand-pivot-sit transfer, standing with balance perturbation, curb or stair climbing, and walking [3–9].

Muscle strength training was effective in improving muscle strength following stroke [10], but conflicting results were showed regarding the transfer effect of lower extremity strength gains on functional outcome [11–13]. Contemporary principle of stroke rehabilitation put emphasis on task-oriented training [14]. Recent study showed that task-oriented progressive resistance strength training program could improve lower extremity muscle strength in individuals with chronic stroke and could carry over into improvement in functional abilities [15]. TA muscle exercise training with task-oriented training is an essential part of stroke rehabilitation; however, it is difficult to execute exercise training for the TA muscle due to substantial weakness and insufficient muscle recruitment in patients with different severities [16]. Hence, evidence supporting the effects of TA muscle exercise training on enhancing strength or improving lower limb motor function is scarce in the stroke literature [9, 17].

Electromyographic biofeedback (EMGBFB) has been recommended as a good adjunct tool for stroke rehabilitation to help muscle training and enhance motor relearning by providing visual or audio feedback of muscle activation [14, 18]. However, the evidence on EMGBFB-assisted exercise training in stroke patients has been inconclusive [19–21]. A few randomized control trials have shown that EMGBFB in combination with standard physical therapy could improve TA muscle strength [22, 23], but recent comprehensive meta-analyses revealed no valid evidence supporting the position that EMGBFB-assisted exercise training could significantly improve motor function such as balance or walking in stroke patients [20, 21]. In addition to the problems with the experimental methodology, the lack of benefits on functional improvement in these trials may have been due to the nonfunctional single muscle contractions in static positions of the training and non-task-oriented training mode [24]. Aiello and associates developed a treadmill training system combined with TA and gastrocnemius lateralis (GA) muscle EMGBFB and found temporary improvements in both ankle muscle power and gait function in stroke patients [25]. There was no control group in this study; furthermore, the clinical implication for using a dynamic EMGBFB-assisted treadmill system is limited, since most clinicians lack access to this type of training instrument. Jonsdottir and associates used a portable EMGBFB machine, which is popular in clinics, to provide GA muscle feedback and to assist overground gait training for chronic stroke patients [26]. After 20 sessions of walking-related EMGBFB-assisted exercise training, positive long-term effects were demonstrated in ankle power and walking ability comparing with usual rehabilitation care. Based on the review of the above trials, EMGBFB that was applied with task-related activities might effectively improve both muscle strength and motor task for chronic stroke compared with usual physical therapy group.

In addition to the application of a current motor learning concept to the EMGBFB-assisted exercise training program by utilizing the task-related training mode, variation of practice is also a critical factor for effective motor training [14, 18, 27]. Although there is evidence from unimpaired populations that variable practice can be more advantageous than constant practice to enhance the ability to adapt and generalize motor learning that leads to better performance in novel tasks, there were few studies that investigated the effect of different types of practice in people with stroke [28–34]. We suspect that the effects on muscle strength and motor function of EMGBFB-assisted TA exercise training with a portable system may be enhanced if practice variation is added into the exercise program. Contemporary principles of stroke rehabilitation are based on motor learning paradigms and should include meaningful, repetitive, intensive, and task-specific movement training with feedback along with practice manipulations to promote neural plasticity and motor recovery. Task-related EMGBFB exercise training, in which a muscle training program is conducted in task-related activities with faded feedback and practice variation, would likely maximize the motor function recovery. For rehabilitation of chronic stroke, it is important to develop an effective and feasible clinical training strategy for patients suffering from impaired TA muscle force control. The purpose of this study was to determine the effects of constant force or variable force practice with task-related EMGBFB-assisted TA exercise training, according to the principles of motor learning, on the TA muscle strength, balance, and lower limb motor function in people with chronic stroke.

## 2. Materials and Methods

This was an assessor-blinded, randomized, controlled trial. Participants were recruited from the National Taiwan University Hospital in Taiwan.

The inclusion criteria were (1) age more than 18 years old, (2) unilateral stroke for more than 3 months, (3) active ankle dorsiflexion angle of the affected side less than that of the less-affected side, (4) ability to stand for more than 20 seconds independently, (5) ability to walk 10 meters with or without devices, and (6) ability to comprehend and follow verbal instructions for the motor tasks and tests in this study. The exclusion criteria were (1) presence of Parkinsonism or other neurological system diseases, (2) knee or hip arthroplasty or recent lower extremity pain (within 1 month), and (3) recurrent stroke. All participants gave written informed consent, and an ethics committee approved this study.

### 2.1. Procedures

**Figure 1:**
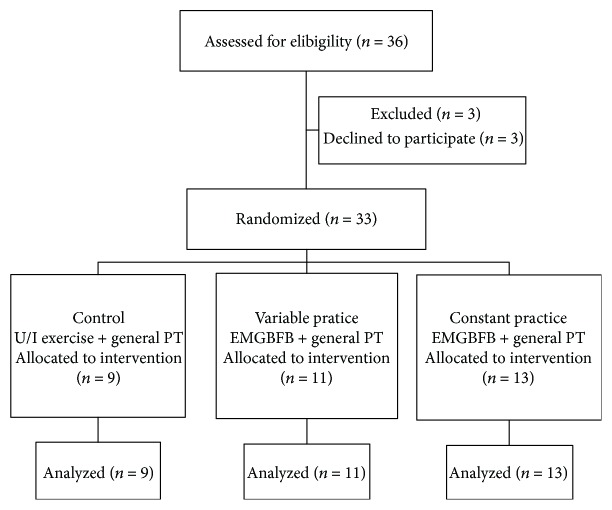
The procedure for this study.

The procedure of the study is presented in Figure 1. Participants were screened for eligibility and then assigned to one of three exercise programs, the constant-force EMGBFB-assisted TA exercise (constant) group, the variable-force EMGBFB-assisted TA exercise (variable) group, or the upper extremity exercise without EMGBFB (control) group, according to computer-generated random numbers.

The assessor was blind to the group allocation of the participants. The trainer and participants were aware of the existence of varied types of exercises, but the participants were unaware of the details of the differences. Each exercise program lasted 6 weeks, during which participants attended 18 sessions of 40 minutes each. The constant and variable groups received EMGBFB-assisted TA exercise training emphasizing constant force or variable force practice, and the control group performed upper extremity exercises without EMGBFB. Assessments were conducted at baseline and at 1 day, 2 weeks, and 6 weeks posttraining by physical therapists blinded to group assignment. In addition to the training provided by the study, all participants received routine outpatient physical therapy offered by the national health insurance 2 to 3 sessions per week. The general physical therapy was a mixture of therapeutic approaches, including neurodevelopmental and neurofacilitation techniques, balance training, gait corrections and treadmill walking exercises, task-specific training, and task-oriented training including upper and lower extremities. No EMFBFB training was conducted during routing general physical therapy. Regardless of group assignment, all participants practiced treadmill walking training routinely.

### 2.2. Intervention

For the constant and variable groups, a portable, 2-channel surface EMGBFB system was used for task-related EMGBFB-assisted TA exercise training. The signal was band-pass filtered at 15 to 300 Hz, and input sensitivity was below 1 *μ*V RMS. A visual or audio signal of EMGBFB was generated from EMG electrodes applied over the affected TA muscle belly after standard skin preparation. The signal was used for feedback of TA muscle contraction performance, and the intensity of signal was positively correlated with the level of muscle contraction.

At the beginning of each training session, the participants performed ankle dorsiflexion while seated in a chair with the hips flexed at 90°, knees flexed at 60°, arms and trunk relaxed, and the affected foot resting on the floor. The maximal EMG signal of the EMGBFB machine during maximal contraction of the affected TA muscle was recorded and used for setting the goal of the current training session. For the constant force practice group, the training goal was to contract the TA muscle to match the maximal EMG signal, i.e., 100% of TA muscle force exertion. For the variable force practice group, the participants were instructed to vary the force outputs of the TA muscle to match EMGBFB signals at 100%, 75%, 50%, or 25% of maximal EMG in a randomized order. Each 40-minute training session was divided into two components, i.e., component I and component II. In component I, TA muscle contractions were practiced in a static seated position and faded EMG feedback was provided. Participants were seated in a chair and practiced 4 blocks of TA muscle contraction trials (holding for 5 seconds per trial with 20 seconds of rest, 20 trials per block, with 2 minutes of rest between blocks) with the heel contacting the floor for a total of 80 trials. Participants had to match the EMG goals during each exercise block, depending on their group assignments. In component II, participants practiced TA muscle contraction during walking and balance-related activities, and faded EMG feedback was also provided. Participants performed EMGBFB-assisted TA exercises during walking and balance-related tasks such as standing with toes up, weight shifting, stepping, going up/down the stairs, or walking, depending on each participant's abilities. The training goals were to contract the affected TA muscle and to match the set EMG goals.

The feedback frequency was faded for both the constant force and the variable force practice groups. The feedback was gradually reduced from 100% (every trial) at the beginning of each daily practice session to 60% (3 within 5 trials), 40% (2 within 5 trials), and then finally to no feedback at the end of the session. The actual number of trials at each feedback frequency was tailored based on the individual's level of performance. That is, the feedback frequency is reduced to the next level when the participant successfully achieved the training target in more than 50% of the previous trials. In the trials with feedback, the sound was turned on and the monitor of the EMGBFB machine was placed to face the participants so that they could read the concurrent EMG traces displayed on the monitor. In the trials without feedback, the sound was turned off and the participants could not read the EMG on the monitor.

Participants in the control group took part in an upper extremity exercise program of range of motion, stretching, and strengthening exercises without EMGBFB. The training schedule was the same as that of the EMG groups. General physical therapy was continued for all three groups.

### 2.3. Outcome Measures

All participants underwent a blinded evaluation of muscle strength, balance, and lower limb motor function at baseline and at 1 day, 2 weeks, and 6 weeks posttraining. The assessors were physical therapists with at least one year of clinical experiences who were familiar with the outcome measures listed below. Before the evaluation, each assessor received at least 8 hours of training to unify details of test and measures for all of the outcome measures used in this study listed below. Baseline participant information including age, gender, height, weight, time since stroke, severity of stroke, affected side, and affected ankle range of motion was collected. Affected ankle range of motion was measured in supine position, while participants were encouraged to dorsiflex their ankle as much as possible. The universal goniometer aligned by fibular head, lateral malleolus, and 5th metatarsal head was used to measure the angel between horizontal plane and maximal ankle dorsiflexion.

#### 2.3.1. Muscle Strength

The strength of the affected TA muscle was measured by a handheld dynamometer with the participant in a sitting posture with the hips flexed at 90° and the knees flexed at 60°. Participants were instructed to dorsiflex the ankle joint as much as possible while the reading from the handheld dynamometer was recorded. The TA muscle strength was measured three times, and the average value (in Newton (NT)) was used for further analysis.

#### 2.3.2. Balance

The Smart Balance Master System was used to evaluate dynamic balance with the limit of stability (LOS) test. The LOS test examines the ability of a standing participant to lean the body in 8 directions as quickly and accurately as possible. Only the anteroposterior direction was included in the final analysis because the main function of the TA muscle is to control movement in this direction [35] and also because our previous study showed trends of improvement only in this direction [36]. The measured parameter of the LOS test was the endpoint excursion (EPE), which is the distance of the first movement toward the designated target expressed as a percentage of maximal LOS distance. 100% LOS indicated that the COG trajectory was a straight line between the center location and the target.

#### 2.3.3. Mobility and Endurance

Mobility- and endurance-related lower limb function was evaluated in this study with walking speed, Timed Up and Go test (TUGT), and six-minute walk test (6MWT). Walking speed was calculated by timing each participant with a stopwatch as they walked at a comfortable speed along a 6 m straight path [37]. The total length of the marked path was 10 m, with 2 m provided at each end for acceleration and deceleration. The TUGT measured the time taken to stand up from a chair of 46 cm in height with arm and back supports, walk forward 3 meters, turn around, walk back to the chair, and sit down [38]. The participants could use assistive devices and wore the same shoes for all the tests. The 6MWT tested the maximal distance covered during walking for 6 minutes [39]. To prevent fatigue, the 6MWT was performed only once, and each of the other tests was practiced once to warm up and then performed three times. The average score was recorded for analysis.

### 2.4. Data Analysis

The intraclass correlation coefficient (ICC) was used to examine the interrater reliability between different assessors performing TA muscle strength, walking speed, and TUGT. All data were analyzed with the SPSS 17.0 software. Repeated-measures analysis of variance (repeated ANOVA) was used to assess the effects of groups (constant, variable, and control) and testing sessions (at baseline and at 1 day, 2 weeks, and 6 weeks posttraining) on parametric motor performance outcomes such as TA muscle strength, walking speed, TUGT, and 6MWT. If the interaction effect was detected, post hoc analysis was conducted using Tukey's test. The change scores were calculated by subtracting the baseline data from the posttraining data and were used to analyze the intergroup effects. All outcomes were analyzed using an intention-to-treat analysis with imputation for missing data, as last observation carried forward. For all statistical tests, significance was set at *P* < 0.05.

## 3. Results

Thirty-three participants completed this study (constant *n* = 13; variable *n* = 11; control *n* = 9). The mean age was 53.33 ± 11.78 years (range, 26–74 years; male *n* = 26, female *n* = 7). Three participants did not complete the training because of transportation difficulties. The demographic and clinical characteristics of the participants in the three groups are shown in Table 1. All participants were right-side dominant. There were more right sides affected in the variable group. Also, age and time since stroke appear to be lower for the variable group. But no significant differences in the demographic and clinical characteristics were found between the groups at baseline. All participants completed the study exercise protocol, and the attendance rate was 100%. A total of five assessors participated in this study. The interrater reliabilities (ICC) of TA muscle strength, walking speed, and TUGT were 0.990, 0.976, and 0.993, respectively.

**Figure 2:**
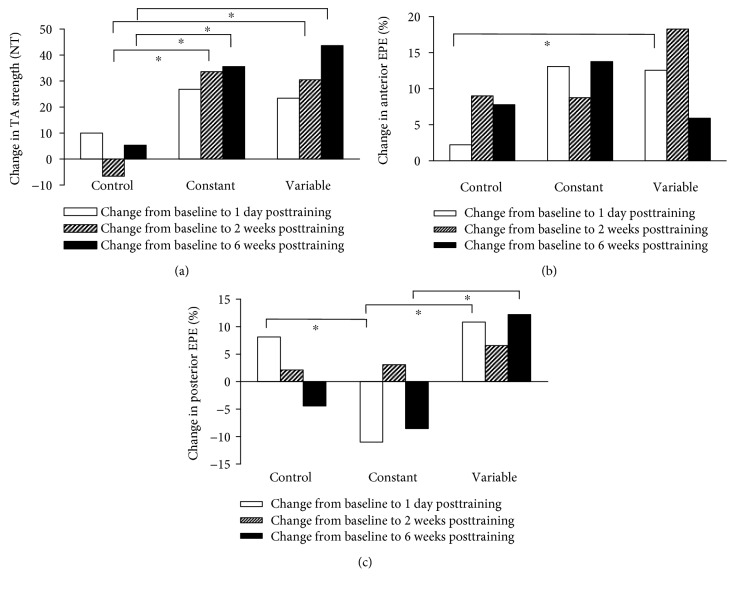
Changes in TA strength and balance after training. (a) Change in TA strength. (b) Change in anterior EPE. (c) Change in posterior EPE. ^∗^Requirement for a statistically significant difference: *P* < 0 05.

Table 2 shows the motor performance data, including affected TA muscle strength, anterior EPE, posterior EPE, walking speed, TUGT, and 6MWT, in the three groups during the four testing sessions. There were no significant differences between any of the measures at baseline. Repeated-measures analysis revealed significant interaction effects between testing sessions and groups for the strength of the affected TA muscle (*P* = 0.015). Post hoc pairwise comparisons revealed that the affected TA muscle strength was significantly higher in both EMGBFB groups at 2 weeks posttraining and 6 weeks posttraining than at baseline (Figure 2(a)). No significant difference was found between the constant and variable groups. There was no significant change in affected TA muscle strength in the control group from the baseline to 1 day posttraining, 2 weeks posttraining, or 6 weeks posttraining.

Balance function was indicated by the EPE in both anterior and posterior directions. The nonparametric statistical test revealed that the two directions of EPE in the control group did not show a significant difference in the intragroup comparison. The anterior EPE in the constant group presented a significant increase in the intragroup comparison from baseline to the three posttraining testing sessions (from baseline to 1 day posttraining, *P* = 0.016; from baseline to 2 weeks posttraining, *P* = 0.049; and from baseline to 6 weeks posttraining, *P* = 0.003), but the anterior EPE did not differ in the intergroup comparison. The posterior EPE in the constant group showed a significant decrease between testing sessions from baseline to 1 day posttraining (*P* = 0.035) and from baseline to 6 weeks posttraining (*P* = 0.035). In the constant group, the posterior EPE decreased significantly from baseline to 1 day posttraining, as compared with the control group (*P* = 0.033) (Figure 2(c)). In the variable group, the anterior EPE significantly increased in the intragroup comparison from baseline to 1 day posttraining (*P* = 0.003) and from baseline to 2 weeks posttraining (*P* = 0.013). In the variable group, anterior EPE increased significantly from baseline to 1 day posttraining as compared with the control group (*P* = 0.034) (Figure 2(b)). The posterior EPE in the variable group increased significantly in the intragroup comparison from baseline to 6 weeks posttraining (*P* = 0.018). In the intergroup comparison between the constant and variable groups, the posterior EPE increased significantly in the variable group from baseline to 1 day posttraining (*P* = 0.008) and from baseline to 6 weeks posttraining (*P* = 0.004) (Figure 2(c)). Repeated-measures analysis did not show any significant effects for walking speed, TUGT, or 6MWT between the groups or testing sessions.

## 4. Discussion

Our study demonstrated that the combined use of a portable EMGBFB system and leg (TA) muscle active exercises, administered during task-related activities and following motor learning principles, improved TA muscle strength in patients with chronic stroke. Increases in TA muscle strength persisted for up to 6 weeks after training. Practicing variable force output with augmented feedback from the EMGBFB during task-related movement improved the training effects to the balance function. However, our results did not demonstrate a significant improvement of training effects to walking speed, TUGT, or 6MWT in the three groups.

The participants in both the variable force practice and constant force practice EMGBFB-assisted TA exercise groups showed significant improvements in muscle strength on the affected side after 18 sessions (6 weeks) of training. Previous nonfunctional EMGBFB training, which applied in static postures and not as part of functional movement activities, yielded limited benefits on muscle strength and motor function [19–21]. Task-oriented training and motor learning principles have been advocated to maximize the effects of EMGBFB training [14, 24, 26]. In view of this demand, sophisticated EMGBFB systems builds specifically for research have demonstrated support for a task-oriented approach with inclusion of EMGBFB-assisted training of the TA muscles to improve strength and gait of stroke patients. Our study tried to duplicate the good effect of task-oriented EMGBFB training on stroke subjects using a commercially available, portable EMGBFB system. Our EMGBFB training program was conducted during task-related activities and focused on varying the speed and range of ankle movement. Furthermore, based on suggestions from the motor learning literature, faded feedback was provided. The positive results of our study support the current opinions that task-oriented and motor learning principles should be employed to maximize training effects during rehabilitation and that training effects can be demonstrated after shorter episodes of training. Although strengthening exercises are often difficult to execute for stroke patients due to substantial weakness of the TA muscle [16], EMGBFB offers augmented feedback on muscle contractions, which helps stroke patients to recognize successful TA muscle contractions [14]. It also enables patients to practice directly and repeatedly and to gain pleasure and a sense of achievement from their own intended actions [14]. EMGBFB reinforces the strength training effects for stroke patients who have regained some degree of TA muscle force control. Thus, our study supports previous findings that EMGBFB-assisted TA training is effective in improving TA muscle strength in stroke patients. We further revealed that employing the principles of task-oriented and motor learning with a portable and clinically feasible EMGBFB instrument may enhance the training effects in patients with chronic stroke who have impaired TA muscle strength.

Practice variation is one of the important factors for enhancing motor performance and learning in stroke. Evidence has shown that variable practice induces the ability to adapt and the generalization of motor learning to a greater degree than constant force practice does [31]. Repetitions of an action are required to increase muscle strength and limb control and also to develop an optimal way of performing the action [14]. “Repetition without repetition” means that repeating an action with varying locations, conditions, or goals can help individuals to develop the ability to solve motor problems and acquire motor skills [14]. Our previous study found that constant force practice EMGBFB training of TA muscles failed to significantly improve the ability of active weight shifting in the anterior and posterior directions [36]. In the current study, the variable force practice component was added, and participants practiced TA muscle contractions at varied extents of muscle force outputs in random order, which simulated the concept of repetition without repetition. Our constant force practice group, on the other hand, practiced TA muscle contractions to 100% force output on each trial, which made their training more like drills, or repetition without variation. One would expect the training effects to daily motor function to be more prominent in the variable force practice group than in the constant force practice group. The present study supports this hypothesis in that balance function significantly improved only in the variable force practice group and not in the constant force practice group. The ability of a standing participant to lean the body in the anterior-posterior direction clearly improved. Past research by Jonsdottir and colleagues presented similar positive results after task-oriented EMGBFB-assisted training on the GA muscle with practice variation, which transferred the training effects to gait [26]. However, their study did not investigate balance function. In our study, we further recognized the influences of practice variation on motor function recovery after EMGBFB-assisted exercise training for stroke patients. Thus, EMGBFB-assisted TA exercise training with variable force practice is more effective than that with constant force practice for the improvement of balance function.

The present study has several limitations. First, the dosage (12 hours) of our treatment was smaller than some of the other task-oriented programs which demonstrated significant improvement in people with stroke, such as the task-oriented EMGBFB training study by Jonsdotti and associates (15 hours) [26], meaningful task-specific training on the upper extremity motor recovery by Arya and associates (20 hours) [40], and constraint-induced movement therapy on upper extremity function by Wolf and colleagues (60 hours) [41]. Our selection of 12 hours was based on the visual EMGBFB study by Aiello and colleagues [25] and has doubled from our previous study using just 6 hours EMGBFB training for chronic stroke [36]. Thus, even though we have successfully demonstrated that the effects of EMG-assisted TA muscle training significantly improved the TA muscle strength, the effects of constant vs. variable conditions might have been more different with increased practice hours. Second, our results showed significant improvements in all three groups in walking speed, TUGT, and 6MWT. This might be related to the fact that all subjects received routine treadmill training and gait correction physical therapy programs. Treadmill training has been found to effectively enhance walking-related outcomes such as walking speed and 6MWT [42]. Thus, our training effects may have been masked by this strong influence in walking-related outcomes. Third, our choice of a comfortable walking speed, rather than a maximal walking speed, as the outcome measure may have lessened the possibility of demonstrating mild training effects that could have been demonstrated in a more strenuous and sensitive measurement protocol. It has been suggested that fast walking speed reflects the ability to voluntarily increase walking velocity better than a comfortable speed test [43]. We suggest that a fast walking speed test be added in future studies of EMGBFB-assisted TA exercise training. Fourth, whether the EMGBFB boosted the effects of a pure TA muscle training program remains unanswered. Our participants in the control group took part in an upper extremity exercise program without EMGBFB. This design could investigate the effects of combined use of EMGBFB and leg exercises during task-oriented activities compared to upper extremity training, not to compare between the effects of a pure TA muscle exercise and EMGBFB-assisted TA exercise training. It is needed to conduct the pure TA muscle exercise without EMGBFB for the control group in further study. Fifth limitation was uneven about right or left affected side, age, and time since stroke between groups. There were apparently more right sides affected, younger, and less time since stroke for the variable group participants. Although no significant difference was presented, dominant side affected, younger, or shorter time since stroke would be contributed to more improvement. Further study may need to control these factors by using a stratified group design.

## 5. Conclusions

Task-related EMGBFB-assisted TA exercise training helped to improve affected TA muscle strength in chronic stroke participants. Practicing variable force with augmented faded feedback from the EMGBFB during task-related movement improved the balance function. The muscle strength effects persisted up to 6 weeks posttraining, indicating that this training regimen was effective and feasible for people having impaired TA muscle strength. Our study highlights the importance of task-related training and motor learning principles in EMGBFB training. EMGBFB-assisted exercise training in task-related activities with faded feedback and practice variation is recommended for patients with chronic stroke and TA muscle force insufficiency.

## Figures and Tables

**Table 1:** Demographic and clinical characteristics of participants in the three groups.

	Control (*n* = 9)	Constant (*n* = 13)	Variable (*n* = 11)	*P*
Age (years)	56.1 ± 9.0 (42–66)	55.5 ± 12.4 (29–74)	48.6 ± 12.6 (26–68)	0.26
Gender (M/F)	7/2	9/4	10/1	0.49
Height (cm)	167.3 ± 5.9 (158–175)	164.8 ± 9.0 (148–178)	166.9 ± 6.6 (153–175)	0.68
Weight (kg)	60.9 ± 7.0 (45–68)	64.9 ± 12.0 (46–85)	66.7 ± 11.9 (51–88)	0.49
Time since stroke (month)	14.8 ± 8.7 (4–28)	15.85 ± 11.1 (4–42)	11.6 ± 10.7 (3–41)	0.60
Severity (Br.)	4.7 ± 0.5 (4-5)	4.5 ± 0.7 (3–5)	4.6 ± 0.7 (3–5)	0.70
Affected side (R/L)	4/5	7/7	9/2	0.22
Affected ankle range of motion	69.48 ± 9.44 (51.67–83)	66.13 ± 14.04 (42.67–84.67)	73.73 ± 16.02 (54.33–101)	0.62

Values are mean ± standard deviation (range). Abbreviations: M/F: male/female; Br.: Brunnstrom stage of lower extremity; R/L: right/left.

**Table 2:** Motor performance data at baseline and after training.

Variables	Groups	Baseline	1 day posttraining	2 weeks posttraining	6 weeks posttraining
TA muscle strength (NT)	Control	118.31 ± 61.42	128.31 ± 61.19	111.69 ± 55.75	117.55 ± 40.48
	Constant	102.01 ± 49.66	128.87 ± 62.51^∗^	135.63 ± 56.39^∗^	137.96 ± 67.05^∗^
	Variable	136.53 ± 49.93	159.97 ± 55.98^∗^	167.05 ± 54.55^∗^	189.28 ± 55.82^∗^

Anterior EPE (%)	Control	35.22 ± 15.60	37.44 ± 9.70	44.22 ± 27.69	43.00 ± 13.86
	Constant	35.62 ± 16.35	48.69 ± 17.30^∗^	44.38 ± 17.35^∗^	49.38 ± 19.88^∗^
	Variable	38.18 ± 11.62	50.73 ± 11.47^∗^	56.45 ± 20.67^∗^	44.09 ± 12.59

Posterior EPE (%)	Control	36.44 ± 9.06	44.56 ± 17.85	38.56 ± 10.76	32.00 ± 20.32
	Constant	37.15 ± 15.65	26.15 ± 21.39^∗^	40.23 ± 15.16	28.62 ± 14.60^∗^
	Variable	32.73 ± 15.49	43.55 ± 13.64	39.27 ± 11.24	44.91 ± 9.08^∗^

Walking speed (m/sec)	Control	0.57 ± 0.29	0.65 ± 0.32	0.69 ± 0.31	0.67 ± 0.30
	Constant	0.57 ± 0.25	0.61 ± 0.26	0.64 ± 0.28	0.69 ± 0.29
	Variable	0.71 ± 0.29	0.76 ± 0.30	0.75 ± 0.27	0.74 ± 0.26

TUGT (sec)	Control	26.50 ± 14.90	24.74 ± 15.38	24.23 ± 14.99	22.77 ± 15.12
	Constant	24.08 ± 8.95	21.84 ± 9.11	20.81 ± 7.73	20.26 ± 10.55
	Variable	20.68 ± 11.19	19.39 ± 9.58	18.09 ± 7.90	17.19 ± 6.56

6MWT (m)	Control	190.11 ± 106.58	214.06 ± 109.94	223.44 ± 104.86	222.67 ± 104.17
	Constant	198.08 ± 96.62	222.77 ± 89.39	232.31 ± 99.11	242.62 ± 109.87
	Variable	227.55 ± 94.60	235.27 ± 89.40	238.64 ± 85.65	250.82 ± 93.81

Values are the mean ± standard deviation. Abbreviations: TA: tibialis anterior; NT: Newton; AROM: active range of motion; m/sec: meter/second; TUGT: Timed Up and Go test; sec: second; 6MWT: six-minute walking test; m: meter; EPE: endpoint excursion. Intragroup: ^∗^*P* < 0.05, when compared with baseline.

## Data Availability

The data used to support the findings of this study are available from the corresponding author upon request.

## References

[1] Lawrence E. S., Coshall C., Dundas R. (2001). Estimates of the prevalence of acute stroke impairments and disability in a multiethnic population. *Stroke*.

[2] Adams R. W., Gandevia S. C., Skuse N. F. (1990). The distribution of muscle weakness in upper motoneuron lesions affecting the lower limb. *Brain*.

[3] Knutsson E., Richards C. (1979). Different types of disturbed motor control in gait of hemiparetic patients. *Brain*.

[4] Arene N., Hidler J. (2009). Understanding motor impairment in the paretic lower limb after a stroke: a review of the literature. *Topics in Stroke Rehabilitation*.

[5] Beaulieu L. D., Masse-Alarie H., Brouwer B., Schneider C. (2014). Brain control of volitional ankle tasks in people with chronic stroke and in healthy individuals. *Journal of the Neurological Sciences*.

[6] Cheng P. T., Chen C. L., Wang C. M., Hong W. H. (2004). Leg muscle activation patterns of sit-to-stand movement in stroke patients. *American Journal of Physical Medicine & Rehabilitation*.

[7] Dorsch S., Ada L., Canning C. G., Al-Zharani M., Dean C. (2012). The strength of the ankle dorsiflexors has a significant contribution to walking speed in people who can walk independently after stroke: an observational study. *Archives of Physical Medicine and Rehabilitation*.

[8] Fujimoto M., Hsu W. L., Woollacott M. H., Chou L. S. (2013). Ankle dorsiflexor strength relates to the ability to restore balance during a backward support surface translation. *Gait & Posture*.

[9] Bohannon R. W. (2007). Muscle strength and muscle training after stroke. *Journal of Rehabilitation Medicine*.

[10] Morris S. L., Dodd K. J., Morris M. E. (2004). Outcomes of progressive resistance strength training following stroke: a systematic review. *Clinical Rehabilitation*.

[11] Sharp S. A., Brouwer B. J. (1997). Isokinetic strength training of the hemiparetic knee: effects on function and spasticity. *Archives of Physical Medicine and Rehabilitation*.

[12] Weiss A., Suzuki T., Bean J., Fielding R. A. (2000). High intensity strength training improves strength and functional performance after stroke. *American Journal of Physical Medicine & Rehabilitation*.

[13] Ouellette M. M., LeBrasseur N. K., Bean J. F. (2004). High-intensity resistance training improves muscle strength, self-reported function, and disability in long-term stroke survivors. *Stroke*.

[14] Carr J. H., Shepherd R. B., Carr J. H., Shepherd R. B. (2010). Training motor control, increasing strength and fitness and promoting skill acquisition. *Neurological Rehabilitation: Optimizing Motor Performance*.

[15] Yang Y. R., Wang R. Y., Lin K. H., Chu M. Y., Chan R. C. (2006). Task-oriented progressive resistance strength training improves muscle strength and functional performance in individuals with stroke. *Clinical Rehabilitation*.

[16] Dragert K., Zehr E. P. (2013). High-intensity unilateral dorsiflexor resistance training results in bilateral neuromuscular plasticity after stroke. *Experimental Brain Research*.

[17] Hammami N., Coroian F. O., Julia M. (2012). Isokinetic muscle strengthening after acquired cerebral damage: a literature review. *Annals of Physical and Rehabilitation Medicine*.

[18] Boddice G. B. S., Gustafsson L., Kenardy J., Hoffmann T. (2010). *Clinical Guidelines for Stroke Management 2010*.

[19] Moreland J. D., Thomson M. A., Fuoco A. R. (1998). Electromyographic biofeedback to improve lower extremity function after stroke: a meta-analysis. *Archives of Physical Medicine and Rehabilitation*.

[20] Woodford H., Price C. (2007). EMG biofeedback for the recovery of motor function after stroke. *Cochrane Database of Systematic Reviews*.

[21] Veerbeek J. M., van Wegen E., van Peppen R. (2014). What is the evidence for physical therapy poststroke? A systematic review and meta-analysis. *PLoS One*.

[22] Burnside I. G., Tobias H. S., Bursill D. (1982). Electromyographic feedback in the remobilization of stroke patients: a controlled trial. *Archives of Physical Medicine and Rehabilitation*.

[23] Basmajian J. V., Kukulka C. G., Narayan M. G., Takebe K. (1975). Biofeedback treatment of foot-drop after stroke compared with standard rehabilitation technique: effects on voluntary control and strength. *Archives of Physical Medicine and Rehabilitation*.

[24] Huang H., Wolf S. L., He J. (2006). Recent developments in biofeedback for neuromotor rehabilitation. *Journal of Neuroengineering and Rehabilitation*.

[25] Aiello E., Gates D. H., Patritti B. L. Visual EMG biofeedback to improve ankle function in hemiparetic gait.

[26] Jonsdottir J., Cattaneo D., Recalcati M. (2010). Task-oriented biofeedback to improve gait in individuals with chronic stroke: motor learning approach. *Neurorehabilitation and Neural Repair*.

[27] Shumway-Cook A., Woollacott M. H. (2007). *Motor Control: Translating Research into Clinical Practice*.

[28] Cohen H. S., Bloomberg J. J., Mulavara A. P. (2005). Obstacle avoidance in novel visual environments improved by variable practice training. *Perceptual and Motor Skills*.

[29] Douvis S. J. (2005). Variable practice in learning the forehand drive in tennis. *Perceptual and Motor Skills*.

[30] Memmert D. (2006). Long-term effects of type of practice on the learning and transfer of a complex motor skill. *Perceptual and Motor Skills*.

[31] King A. C., Newell K. M. (2013). The learning of isometric force time scales is differentially influenced by constant and variable practice. *Experimental Brain Research*.

[32] Yao W. X., Cordova A., De Sola W., Hart C., Yan A. F. (2012). The effect of variable practice on wheelchair propulsive efficiency and propulsive timing. *European Journal of Physical and Rehabilitation Medicine*.

[33] Catalano J. F., Kleiner B. M. (1984). Distant transfer in coincident timing as a function of variability of practice. *Perceptual and Motor Skills*.

[34] McCracken H. D., Stelmach G. E. (1977). A test of the schema theory of discrete motor learning. *Journal of Motor Behavior*.

[35] Moore S. P., Rushmer D. S., Windus S. L., Nashner L. M. (1988). Human automatic postural responses: responses to horizontal perturbations of stance in multiple directions. *Experimental Brain Research*.

[36] Tsaih P. L., Hu M. H., Shih Y. L., Lin K. H. (2006). Can low-intensity electromyographic biofeedback training on tibialis anterior improve strength and balance in chronic stroke. *FJPT*.

[37] Goldie P. A., Matyas T. A., Evans O. M. (1996). Deficit and change in gait velocity during rehabilitation after stroke. *Archives of Physical Medicine and Rehabilitation*.

[38] Podsiadlo D., Richardson S. (1991). The timed “Up & Go”: a test of basic functional mobility for frail elderly persons. *Journal of the American Geriatrics Society*.

[39] Butland R. J., Pang J., Gross E. R., Woodcock A. A., Geddes D. M. (1982). Two-, six-, and 12-minute walking tests in respiratory disease. *British Medical Journal (Clinical Research Ed.)*.

[40] Arya K. N., Verma R., Garg R. K., Sharma V. P., Agarwal M., Aggarwal G. G. (2012). Meaningful task-specific training (MTST) for stroke rehabilitation: a randomized controlled trial. *Topics in Stroke Rehabilitation*.

[41] Wolf S. L., Winstein C. J., Miller J. P. (2006). Effect of constraint-induced movement therapy on upper extremity function 3 to 9 months after stroke: the EXCITE randomized clinical trial. *JAMA*.

[42] Polese J. C., Ada L., Dean C. M., Nascimento L. R., Teixeira-Salmela L. F. (2013). Treadmill training is effective for ambulatory adults with stroke: a systematic review. *Journal of Physiotherapy*.

[43] Dobkin B. H. (2006). Short-distance walking speed and timed walking distance: redundant measures for clinical trials?. *Neurology*.

